# Pituitary adenylate-cyclase-activating polypeptide (PACAP): another novel target for treatment of primary headaches?

**DOI:** 10.1186/s10194-018-0860-4

**Published:** 2018-05-08

**Authors:** Messoud Ashina, Paolo Martelletti

**Affiliations:** 1 Department of Neurology, Danish Headache Center, Rigshospitalet Glostrup, Faculty of Health and Medical Sciences, University of Copenhagen, Copenhagen, Denmark; 2 Department of Clinical and Molecular Medicine, Sapienza University, Rome, Italy; 3 Regional Referral Headache Centre, Sant’Andrea Hospital, Rome, Italy

Since its discovery in 1989 [1], pituitary adenylate cyclase activating peptide (PACAP) has emerged as a key molecule in primary headaches. New insights on the role of PACAP in migraine [2] have now been translated into interest in exploring the migraine preventative effect of monoclonal antibodies against PACAP and monoclonal antibodies against PAC1 receptor (https://clinicaltrials.gov/ct2/show/NCT03238781?term=AMG301&rank=1 ;[3]).

Given that PACAP is a multifunctional peptide involved in various cellular and physiological responses, it is important to understand and further explore its function in non-headache mechanisms which might be relevant in context to primary headaches. This TJHP thematic series provide an overview of the history of the discovery of PACAP and its three receptors [4] and summarize PACAP and its receptor distribution in migraine relevant structures [5]. Furthermore, the readers will get insights on the role of PACAP in regulating the production of inflammatory mediators [6] and important role of degranulation of dural mast cells via a yet unknown receptor in migraine induction by PACAP38 [7]. The series also cover possible mechanisms of PACAP38-induced migraine, outline future directions [8] and discuss how PACAP may cross the blood brain barrier [9] and its implication for PACAP induced migraine. To further elucidate the role of PACAP in primary headaches it is important to study its role in regulation of sleep [10] and vascular tone [11] which are covered by excellent review articles in the special issue Both aspects are important in context of tolerability and safety of future anti PACAP drugs for prevention of primary headaches. Altogether our aim is to provide a comprehensive series of reviews focusing on PACAP as an emerging molecule in primary headache headaches.
